# Polypoid Growth of the Heart

**Published:** 1853-02

**Authors:** J. E. Nield

**Affiliations:** Resident Medical Officer


### PATHOLOGY AND PRACTICE OF MEDICINE

*Polypoid Growth of the Heart.* (Reported by Mr. J. E. Nield, Resident Medical Officer.)—Christopher M——, aged forty-seven, an Irishman, and a discharged soldier on pension; formerly served in India; was admitted as a home-patient of the Rochdale General Dispensary, May 5, 1852. I visited him the same day.

*Symptoms.*—Continuous vomiting of two days' duration, the egesta being streaked with blood; no pain, except during the act of emesis; tongue coated with a dense white fur; pulse rapid and very compressible; alvine functions normal. Ascertaining that a few days previously he had received his pension, and on the strength of it he had been indulging very freely in beer, I judged the case to be one of gastric derangement occasioned by this just-named excess. I sent the following mixture:—Epsom salts, two drachms; hydrocyanic acid, a drachm; camphor mixture, six ounces; half an ounce every three hours. I sent also a small dose of calomel and opium, to be taken when the vomiting should have subsided, as I believed it would.

May 6th.—On visiting the man to-day, I was for the first time informed that on the 3d instant, during his debauch, he became involved in a quarrel with another man, who, in the scuffle, kicked him over the region of the liver. On making pressure over this organ, there was some indication of tenderness, though not so considerable as to suggest serious lesion. There was no external evidence of injury. The vomiting still continued as incessant as before; the pulse was more labored; some dyspnœa had come on. There was no cough, nor other pulmonic symptom beyond the dyspnœa, to draw attention to the condition of the thoracic viscera. The face was much blanched, and the vital powers apparently fast waning, as if from internal hemorrhage. All the phenomena, in fact, pointed to the probability of rupture of the liver. I ordered the most perfect quietude to be observed, and sent a mixture containing small doses of opium. I visited him again in the evening; the pulse was fluttering; the respiration more oppressed; the vomiting persistent; the system merging on collapse. There was also some disturbance of the intellectual powers. I ordered bottles of hot water to the feet and to the epigastrium.

7th.—Nine A. M.: Pulse a mere thread; extremities cold; breathing much oppressed. He died an hour afterwards.

In connexion with Mr. J. E. Wood, one of the staff of this institution, and with his kind and valuable assistance, I made the necropsy, seven hours after death:—

No special external appearances, except a slight contusion on the arch of the nose. The thorax and abdomen being opened, the heart was first examined. The pericardium contained several ounces of serum; the heart itself was somewhat hypertrophied. On being removed from its attachments, a dense fibrinous mass was seen to hang from the severed pulmonary artery; the right ventricle being opened, this body was found to terminate in a lash of fibrinous threads, whose delicate extremities were rather firmly connected to the parietes of this cavity, and to the circumference of the auriculo-ventricular valve. In structure it was firm and elastic, nearly resembling tendon; and to complete the likeness, it was enveloped in a sort of theca, which was jagged and torn in places, as if it had been lately separated from its adhesions to the endocardium. Its length was ten inches; its weight 189 grains. At its superior extremity it was expanded into a leaf-like process. At about half its length, a process was sent off of some thickness. The walls of the ventricle were of more than normal thickness; the endocardium was hyperhæmatous; the valvular structures of the heart were natural; the pleuræ were almost throughout firmly adherent. The lungs were congested, but otherwise healthy. The liver was enlarged, but in structure healthy. *There was no solution of continuity whatever in any part of this viscus.* The stomach was to all appearance healthy, its mucous coat being, if anything, paler than usual; it contained several ounces of yellowish fluid, that had the odor of malt liquor. The rest of the abdominal organs were free from disease, with the exception of the kidneys, which were enlarged and mottled. The blood was remarkably fluid.

*Remarks.*—It is evident that the whole of the phenomena were dependent upon the adventitious formation within the heart. The peculiarity of the symptoms, however, and the fact of previous violence having been experienced, tended altegether to put diagnosis at fault. It cannot be doubted that the polypoid growth had occupied for some time the position in which it was found after death, but that its adhesions had prevented its exercising much influence on the circulating current. Its detachment may be accounted for in two ways—first, by the shock resulting from the blows and falls encountered during the fight—or second, (supposing the sickness to have been consequent on over-imbibition,) by the excessive retching accompanying the vomiting. Perhaps both contributed to this effect. I am less inclined to attribute the vomiting to sympathy with the condition of the heart, inasmuch as when first I visited the patient, I do not remember observing any pulmonic symptoms whatever.

The post-mortem examination was made in obedience to a coroner's order, and the man who had given the kick before spoken of had been placed in custody. I fully believed that the inspection would have disclosed a ruptured liver; and it seemed highly probable that the inquest would terminate in a verdict of manslaughter. The evidence given, however, being of course in accordance with the facts already detailed, the verdict was the usual one of "Died from natural causes."—*Lancet.*

